# Arsenized Devotees Die

**Published:** 1905-09

**Authors:** 


### Arsenized Devotees Die.

It is reported that twelve of the recent deaths from yellow fever have been of adherents of the arsenic theory exploited by Dr. Leach. A formal letter was issued August 22d by Surgeon J. H. White, United States Public Health and Marine Hospital Service, and by the President and Advisory Committee of the New Orleans Medical Society, telling how patients were under treatment who had partaken of the arsenic diet for the supposed sufficient period to render them immune. The letter was drawn forth by the announcement that there would be a mass meeting of the arsenic adherents Wednesday or Thursday night to denounce the medical practitioners of New Orleans.—*Journal A. M. A.*

